# Nurturing Attentiveness: A Naturalistic Observation Study of Personal Care Interactions Between People With Advanced Dementia and Their Caregivers

**DOI:** 10.1093/geront/gnae004

**Published:** 2024-01-24

**Authors:** Tamara Backhouse, Yun-Hee Jeon, Anne Killett, Jessica Green, Mizanur Khondoker, Eneida Mioshi

**Affiliations:** School of Health Sciences, University of East Anglia, Norwich, Norfolk, UK; Sydney Nursing School, The University of Sydney, Sydney, New South Wales, Australia; School of Health Sciences, University of East Anglia, Norwich, Norfolk, UK; School of Health Sciences, University of East Anglia, Norwich, Norfolk, UK; Norwich Medical School, University of East Anglia, Norwich, Norfolk, UK; School of Health Sciences, University of East Anglia, Norwich, Norfolk, UK

**Keywords:** Activities of daily living, Family carers, Long-term care, Personhood, Social care

## Abstract

**Background and Objectives:**

Personal care interactions can provide vital opportunities for caregivers to engage with a person living with advanced dementia. However, interactions may also be a contentious experience, what makes this so is not fully understood. We aimed to examine features of personal care interactions between caregivers and people with advanced dementia to understand how care may be improved.

**Research Design and Methods:**

This was a naturalistic observation study using one-off video-recorded observations of personal care interactions between 14 people with advanced dementia and 12 caregivers (*n* = 7 care-home staff, *n* = 5 family carers) in the United Kingdom (total observation time 03:01:52). Observations were analyzed with observational video coding to determine the frequency of actions of people with dementia and qualitative content analysis for in-depth examination.

**Results:**

Refusals of care were present in 32% of video sections. Active engagement of people with dementia was observed in 66% of sections. Rare *contentious* interactional components were characterized by the person with dementia appearing to show uneasiness and caregivers being flustered and uncertain. However, caregivers typically emanated a nurturing attentiveness, were attuned to the person, and skilled in seamlessly supporting them through care activities.

**Discussion and Implications:**

Findings draw on real-world empirical evidence to reinvigorate the notion of person-centeredness in dementia care. The findings provide much needed insight into practical ways to improve care interactions for people with advanced dementia and enhance their personhood. Appropriate training/guidance for caregivers could support positive personal care experiences for both the person with dementia and caregiver.

## Background

The advanced stages of dementia are often characterized by considerable memory loss; difficulties with mobility, communication, recognition, and behavioral changes (e.g., apathy) due to affected brain regions (Mitchell, 2015). The person may experience incontinence, uncontrollable movements, and difficulties sleeping (Dementia Australia, 2020). Consequently, in the advanced stages, assistance with personal care activities such as washing, eating, and going to the toilet is required (Mitchell, 2015).

Some personal care interactions may not be welcomed by the person with dementia who may refuse assistance with their care. For example, they may say no, turn away, or become angry with the caregiver (Mahoney, 2015). How a person with dementia perceives assistance with their care may relate to multiple factors. For example: (1) their own condition such as impaired cognition, personality and behavioral changes, not understanding caregivers’ intentions, agitation, dependence in activities of daily living, pain, delirium, anxiety, depression, and/or delusions (Backhouse et al., 2022b; Galindo-Garre et al., 2015; Ishii et al., 2012), or (2) caregiver approaches such as communication style, pace, and the techniques and strategies caregivers use to engage the person (Backhouse et al., 2022a; O’Brien et al., 2020; Williams & Herman, 2011). Caregiver approaches can be influenced by other factors, for example, care-home staff may have less time to provide care or to get to know the person well and family carers may be fatigued. Family caregivers may have an advantage, having known the person prior to the development of dementia. However, difficult previous relationships may influence care interactions negatively.

Care interactions where the person with dementia refuses care can be upsetting and stressful for both the person with dementia and their caregiver (Mortensen et al., 2021; Rathnayake et al., 2019). Refusals when there is a clear need for care could also have further negative consequences for the person with dementia such as poor hygiene, urine burns, infections, or loss of the person’s dignity (Backhouse, 2021). Refusals occur in care-home (Backhouse et al., 2020) and family settings (Fauth et al., 2016), across care activities, including teeth cleaning, which is particularly intrusive (Zimmerman et al., 2014), and are frequently targeted for intervention (Backhouse et al., 2020).

Personal care interactions with a person with advanced dementia remain a key source of human contact, communication, and company (Savundranayagam et al., 2016). Skilled caregiver communication, such as being attuned with the person (Alsawy et al., 2017) or providing direct instructions (Stanyon et al., 2019), can be received positively and is pertinent during the advanced stages of dementia where the person’s communication is likely to be impaired (Banovic et al., 2018). However, elderspeak, a form of overaccommodating communication involving inappropriate spoken words and/or intonations for older people, including the use of collective pronouns, the overinclusive “we” or “us” when it should be “you” or “I,” can be perceived negatively by many older people and lead to behaviors that challenge people with dementia (Shaw & Gordon, 2021).

Principles of person-centered care underpin much guidance for dementia care (Brooker, 2003). Person-centered care involves positive person work (Kitwood, 1997): placing the person with dementia at the heart of care, treating them as a unique individual, tailoring care to their needs, valuing their perspectives and rights, and supporting and enabling positive social relationships (Brooker & Latham, 2016). Personhood (subjective and intersubjective psychological well-being) can be upheld through positive interactions and continued engagement (Hennelly et al., 2021). Therefore, using communication components that reflect positive person work can support the person with dementia’s personhood (Mundadan et al., 2023). For example, employing these communication features: *recognition* involving using the person’s name and treating them as an individual; *negotiation* including consulting on preferences and needs; *validation* such as affirming the person and supporting their feelings; and *facilitation* initiating and sustaining interactions (Mundadan et al., 2023).

Person-centered care has become a pervasive concept adopted by many formal dementia care organizations (Brooker, 2003). However, despite almost universal use of the term, it is a nebulous concept interpreted and operationalized in different ways (Byrne et al., 2020) with limited empirical work detailing real-world examples of what person-centered care may look like in practice (Giusti et al., 2020). Person-centered care has been critiqued for focusing on individualistic features, thus ignoring social and relational aspects of personhood (Tieu et al., 2022) and has been used as a politically correct label to reflect the notion of good quality care (Brooker, 2003).

Addressing the person’s need for identity, attachment, and inclusion has been found to improve well-being, whereas actions not upholding comfort, occupation, and inclusion can diminish it (Willemse et al., 2015). Therefore, interactions can enhance or detract from the personhood of the person with dementia (Kitwood, 1997). Rich, meaningful dyadic care interactions have great potential to amplify personhood (Fazio et al., 2018), for example, by providing a sensitive, relational structure (emotional and physical) around the person (Tieu et al., 2022). Socially embedding the person in the activity, taking account of who they are and their preceding life (Tieu et al., 2022), supporting dignity (Fazio et al., 2018), and empowering them as an embodied being (Dewing, 2008) should foster authentic collaboration and a sense of autonomy and belonging (Fazio et al., 2018; Swinton, 2021).

Analyzing real-time observations can enable useful knowledge to be gained about successful ways of communicating and engaging with people with dementia particularly when there are areas of difficulty in the interaction (O’Brien et al., 2020). Our aim was to examine features of personal care interactions between care-home staff and family carers (henceforth collectively termed as caregivers) and people with advanced dementia to understand how care may be improved and inform the development of caregiver educational resources.

## Method

The current study was an exploratory, nonparticipatory, naturalistic observation study (Patton, 2002) of personal care interactions between caregivers and people with advanced dementia with a mixed-methods analysis.

### Setting

The study took place in the East of England, United Kingdom, in four care homes providing accommodation and 24-hr support with personal care for people with dementia, and in five family homes.

### Participants

Participants for this study were people with advanced dementia and their family or care-home caregiver. Eligible people with dementia were aged 65 or older with advanced dementia. Those eligible as having advanced dementia were assessed as being in the severe, very severe, or profound stages of dementia using the Frontier Dementia Rating Scale (FRS). The FRS is a well-validated, informant-based interview, staging tool assessing features such as self-care, motivation, behaviors, and participation in daily activities (Mioshi et al., 2010). The first author, an experienced care-home worker and postdoctoral dementia care researcher, conducted the assessment after consent. Eligible caregivers were providing physical assistance with personal care to the person with advanced dementia. Family carers were primary carers for the person and care-home staff provided personal care for the person at least six times per week.

### Recruitment

This research was linked to a parent study (Pro-CARE) in England, which was funded by the Alzheimer’s Society and used questionnaires to determine factors associated with refusals of care in dementia (Backhouse et al., 2022b). Participants in the parent study (260 participants; 130 people with advanced dementia and 130 caregivers [family or care home]) had the choice to opt-in to take part in video-recorded observations. Eighty-one participants (16 care-home staff, 19 care-home residents, 23 family caregivers, 23 people with dementia supported at home) opted into the observation study. To maximize learning from those who had opted into the observations, maximum variation purposive sampling (Etikan et al., 2016) took place. Dimensions of variability were those who, in the parent study, had reported refusals of care occurred and those who had not, and those from different care homes. We selected and collected data from 26 participants; seven care-home staff, nine care-home residents, five family caregivers, and five people with dementia supported at home before the coronavirus disease 2019 pandemic stopped further selection and data collection from any of the remaining 55 participants.

#### Care-home staff

Care homes in England provide 24-hr accommodation, food, and assistance with personal care either with or without qualified nursing care. For the parent study, eight care homes in the East of England, identified from the Care Quality Commission database (in the public domain) to be providing specialist care to people living with dementia, were recruited via a letter to the manager, follow-up telephone call, and meeting. Subsequently, written information was distributed to individual care-home staff and residents/family members of residents who were assessed by managers as eligible. Those interested contacted the researcher.

#### Family caregivers

For the parent study, family caregivers were approached in two ways: (1) distribution of information leaflets via community dementia services such as Dementia Cafes and carer support groups; and (2) invitations with written study information were sent to family caregivers who had recorded their interest in taking part in dementia research with either a local dementia research group or a national digital register for dementia research, called Join Dementia Research.

### Ethical Considerations and Consenting Processes

Ethical approval was provided by the Queen’s Square Research Ethics Committee, London (Reference: 251339). Valid consent, or advice, was obtained for all participants. Caregivers were provided with written information sheets; if interested, they discussed the research with the first author and provided written consent. In-line with the Mental Capacity Act (2005) of England and Wales, for people with dementia who lacked the capacity to consent, relatives or close friends were provided with study information and a declaration form and provided advice as to whether they thought the person would have been likely to participate if they had capacity to make the decision. Permission or assent for the researcher to be present in the room and video recording was gained from the caregiver and person with dementia prior to each observation.

### Data Collection

#### Video recordings

Video-recorded observations took place in 2019 and early 2020. Data collection took place in the home of the person with dementia, or care home, in the room where each person’s personal care assistance usually took place (bedroom or bathroom). Care activities were chosen by caregivers as ones they thought the person, and themselves, would not mind having observed and recorded (Table 1 and Supplementary Table A). Observations only took place during activities for which the person receiving assistance was wearing at least underwear that covered their body, such as teeth cleaning and hair washing. Bathing or toilet activities were not observed.

**Table 1.** Sample Characteristics

	Total sample (*n* = 26)	Family caregivers (*n* = 5)	Care-home staff (*n* = 7)	People with dementia supported at home (*n* = 5)	Care-home residents with dementia (*n* = 9)
Female, *n* (%)	20 (77)	5 (100)	7 (100)	3 (60)	5 (56)
Age, average (range)	72 (39–99)	60 (39–74)	57 (49–65)	77 (65–88)	88 (79–99)
Ethnicity, *n* (%)					
White British	25 (96)	4 (80)	7 (100)	5 (100)	9 (100)
White British/Asian	1 (4)	1 (20)	0 (0)	0 (0)	0 (0)
Severity of dementia, *n* (%)					
Severe	9 (64)			2 (40)	7 (78)
Very severe	4 (29)			2 (40)	2 (22)
Profound	1 (7)			1 (20)	0 (0)
Family caregiver relationship to person with dementia, *n*(%)					
Spouse		2 (40)			
Adult child		2 (40)			
Adult child-in-law		1 (20)			
Care-home staff role, *n* (%)					
Senior care assistant			2 (29)		
Care assistant			5 (71)		
Length of time supporting the person-years, average (range)	3.7 (0.5–10)	4.7 (2–10)	2.9 (0.5–7)		
Observation length, minutes	184.5			81.36	103.14
Total (range per dyad)	(02.09–48.25)			(02.09–48.25)	(02.31–25.03)
Observation activities, *n*					
Teeth cleaning	7	3	4		
Beard or hair combing	4	2	2		
Outer dressing	4	2	2		
Face washing	2	1	1		
Nail cutting	5	3	2		
Shaving	4	1	3		
Hearing aids	2	1	1		
Medication	1	1			
Assisted eating	1	1			
Foot spa/feet washing	3		3		
Hair washing	1		1		

The first author video-recorded all observations. A handheld video camera was used with the researcher in the same room as the participants, enabling people with advanced dementia to be aware someone was watching them. No instances of distress due to being observed occurred.

#### Instruments

The Resistance-To-Care Dementia of Alzheimer’s Type (RTC-DAT) scale was used to assess the frequency of different refusal behaviors. This validated scale assesses the duration and intensity of 13 refusal of care behaviors; turn away, pull away, push away, pull/push, grab object, grab person, adduct, hit/kick, say no, cry, threaten, scream/yell, and clench mouth (Mahoney, 2015).

A reduced version of the validated 11-item Menorah Park Engagement Scale (MPES) was used to assess the person with dementia’s engagement in the care activity (Camp et al., 2015). For this study we scored the predominant type of participation (participated, watched, interested in other things, slept/stared into space) and whether anxiety (handwringing, rocking, anxious vocalizations) and pleasure (laugh or smile) were present.

A simple yes or no was recorded as to whether discomfort (wincing or grimacing) was shown within the observation period.

### Analysis

We used a mixed-methods approach to analyze the complexity of personal care interactions at different levels and to expand and strengthen our findings. We conducted observational video coding to determine the frequency of people with dementia’s actions regarding engagement and refusals of care during personal care interactions for each care setting, which provided a context of the interactions to be further explored in qualitative analysis. We employed qualitative content analysis to conduct an in-depth examination of interpersonal dynamics (successful and contentious).

#### Observational video coding

Each recording was split into 5-min sections. End sections with durations less than half of 5 min (2 min, 30 s) were excluded to create equal section sizes. Two researchers (T. Backhouse and J. Green) watched and independently rated the first 5 min of all video-recorded observations; this amounted to 59% of the total 5-min sections (26/44). Cohen’s Kappa scores were calculated to determine interrater reliability. A Cohen score between 0.60 and 0.79 is viewed as moderate and between 0.80 and 0.90 as strong agreement (McHugh, 2012). Disagreements were discussed in relation to each scale’s guidance and observations rewatched until consensus. Cohen’s Kappa scores averaged as strong (mean 0.905) for the RTC-DAT duration items and moderate (mean 0.728) for intensity items, and moderate (mean 0.772) for the MPES (McHugh, 2012). The remaining 41% of the 5-min time sections were assessed by (T. Backhouse) only.

#### Qualitative content analysis

Qualitative content analysis of the whole data set was undertaken (Elo & Kyngas, 2008) with the aim of unpacking real-world care interactions. The framework for the analysis focused on identifying successful and contentious components of care interactions to enable learning to improve care for people with advanced dementia. We defined successful interactional components as those where the caregiver appeared relaxed and the person with dementia was engaged in the care process in some way and not distressed. We defined contentious interactional components as those where the caregiver or person with dementia appeared uncomfortable or anxious, and/or the person with dementia was not engaged in the care process.

Qualitative content analysis involved creating codes, categorizing codes, and then creating generic categories (Elo & Kyngas, 2008). All video recordings were transcribed and anonymized. First, observation recordings were closely watched independently by two researchers (T. Backhouse and J. Green) who made notes on the environment, caregiver actions, person with dementia’s actions, and interaction. A subset of transcripts of the observations were read by two family caregivers and three care-home staff lay advisors who had experience of assisting a person with advanced dementia with their personal care. Guidance and support for lay advisors were provided by the first author. Lay advisors’ thoughts about the content of the transcripts were noted down by the first author, interpretations were discussed and these fed into the analysis. For example, lay advisors highlighted the reassuring nature of caregivers’ actions and instances of guiding people with dementia through care activities. Subsequently, the first author further engaged with all notes and transcripts and team meetings were held to examine transcripts and generate codes portraying successful or contentious interpersonal components of caregiver or recipient actions. The first author then grouped the descriptive codes under tentative higher-level categories that included similar actions and incidents clustered together. These codes and categories were then examined by authors (T. Backhouse, Y.-H. Jeon, A. Killett, E. Mioshi) against data and discussed with conceptual/generic categories created and/or refined over multiple meetings. Regular research team discussions during the data analysis process facilitated rounded interpretations and reduced potential bias. Meanings and interpretations were agreed with all authors.

## Results

### Participants and Observations

Twenty-six separate personal care interactions were video-recorded (total observation time 03:01:52): 12 interactions from five family caregiver/relative with dementia dyads, and 14 interactions from nine care-home staff/resident with dementia dyads, 14 dyads in total. Table 1 shows the characteristics of the sample. Observations took place in four different care homes, with one care-home staff member creating dyads with three different residents in one home. All other participants were part of only one dyad.

### Observational Video Coding

Supplementary Table A shows the type of care interactions observed for each dyad, dispersion of the 44, 5-min observation sections, and scale scores. Refusals of care were present in 32% of observation sections. *Pushing away* was the most common form of refusal in family settings and *saying no* in care-home settings (Supplementary Figure A).

**Figure 1.**
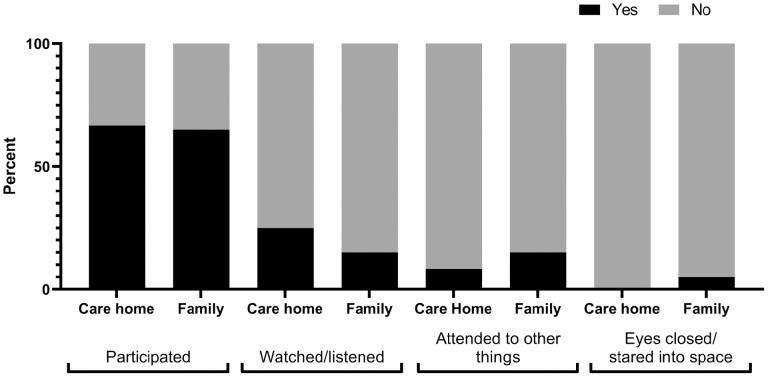
People with dementia’s predominant engagement type during personal care interactions by setting.

**Figure 2.**
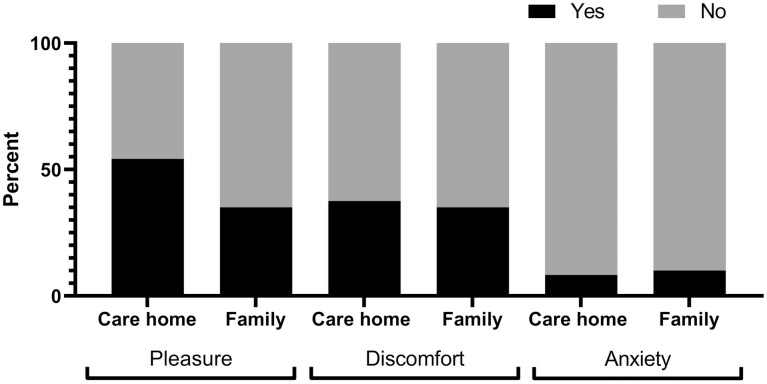
People with dementia’s experiences during personal care interactions by setting.

In 66% of observation sections people with dementia predominantly actively participated in the target care activity (Figure 1). People with dementia expressed pleasure in 54.2% of care-home video sections and 35% of family (Figure 2), showed discomfort in 37.5% of care-home and 35% of family, and showed anxiety in 8.3% of care-home and 10% of family sections.

### Qualitative Content Analysis

**Figure 3.**
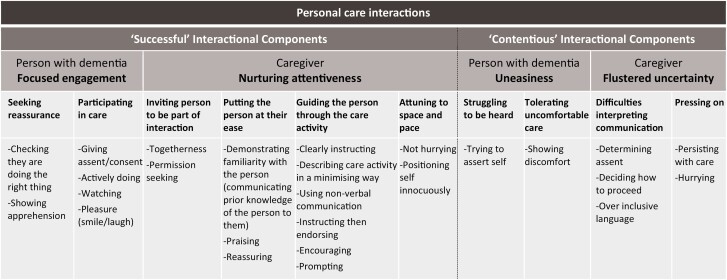
Qualitative content analysis categories.

Figure 3 shows the categories generated through the qualitative analysis. There were two major categories, each with person with dementia and caregiver subcategories. (1) “Successful” interactional components included where the person with dementia showed focused engagement by seeking reassurance and participating in care, and where caregivers showed nurturing attentiveness by inviting the person to be part of the interaction, putting the person at their ease, guiding the person through the care activity, and attuning to place and space. (2) “Contentious” interactional components included where the person with dementia showed uneasiness by struggling to be heard and tolerating uncomfortable care, and where caregivers showed flustered uncertainty through difficulties interpreting communication and pressing on. All categories were present across both family and care-home settings.

Although we observed multiple different care activities (see Table 1), to demonstrate our findings, we use three teeth cleaning observations as exemplars in this paper to consider the findings in depth. As the most intrusive care activity observed, these observations demonstrate all analysis categories.

#### Successful interactional components

Successful components of care interactions were common in both settings and illustrated by the care recipients showing high engagement and caregivers demonstrating a nurturing approach.

##### Example 1: care-home observation

###### Person with dementia’s actions—focused engagement

**Figure 4.**
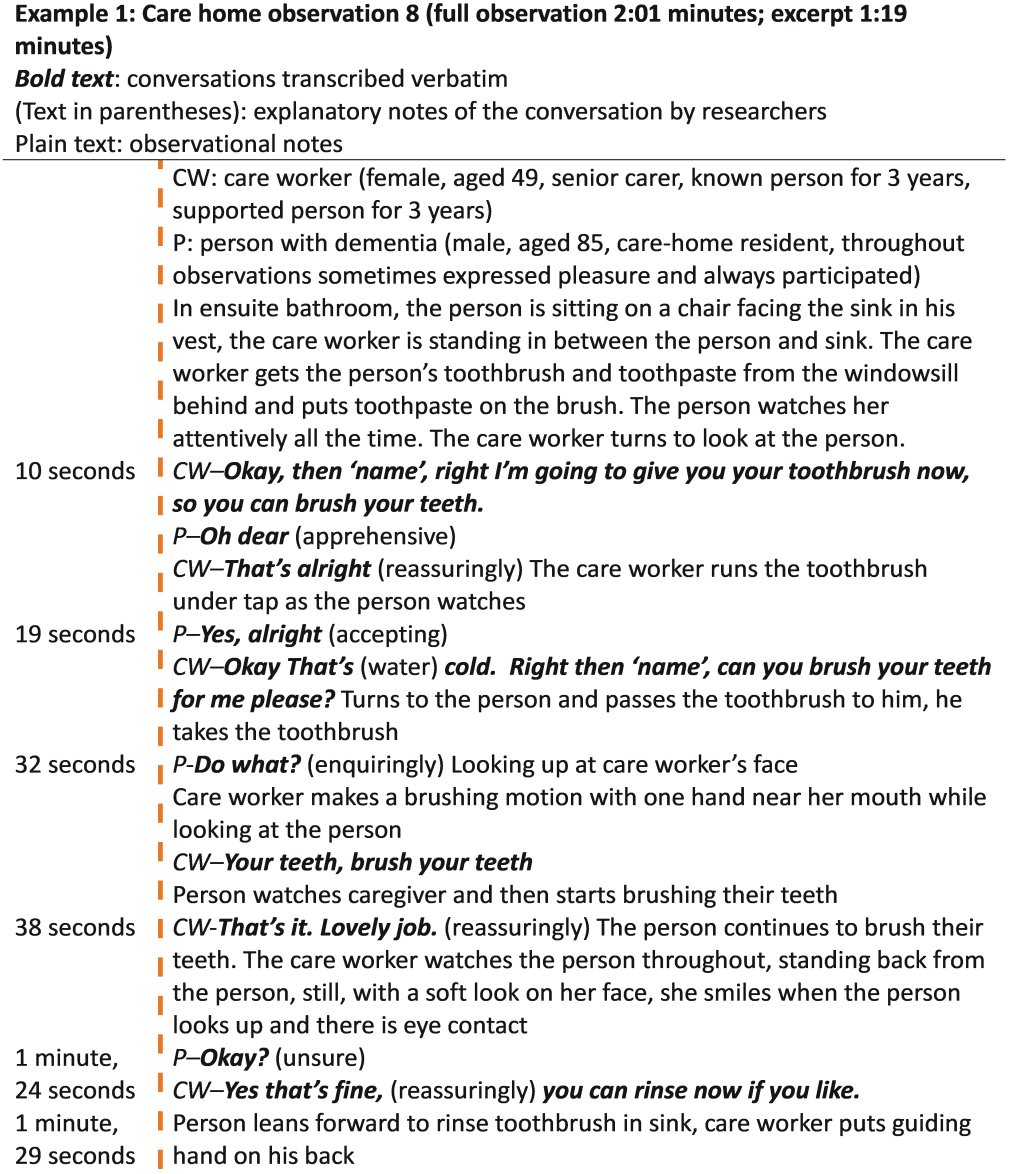
Observation Transcript 1, care-home setting.

Example 1 shows the person was apprehensive in relation to the impending activity (“*oh dear*”; see Figure 4). Consent was provided in response to the caregiver’s invitation and reassurance. The person then sought guidance by checking with the caregiver (“*Do what?*”) when they were not sure of what they had to do. The care-home resident was engaged, actively participating by cleaning their own teeth and when not actively participating, watched the caregiver. The person sought reassurance from the caregiver at the end of the activity (“*Okay?*”).

###### Caregiver’s actions—nurturing attentiveness

The caregiver proposes the activity by using a question (“*Right then ‘name’, can you brush your teeth for me please?*”), including the person’s name and the word *please* to invite them to partake and seek their consent/assent, while also physically passing the toothbrush to the person. In some contexts, the use of “*for me*” could reflect overinclusive speech but from this observation, the language used by the caregiver seemed to elicit encouraging responses from the person with dementia.

Reassurance was provided to the person when they showed apprehension (“*That’s alright*” “*Yes, that’s fine*”). The caregiver used an instruct–endorse pattern (“*Your teeth, brush your teeth*.”–“*That’s it. Lovely job*”): instructing the person to do a task and then endorsing their actions so they were aware they were doing the right thing. This provided the person with dementia with the close nurturing they needed for meaningful engagement.

The caregiver used a nonverbal demonstration (brushing motion with hand) of the action needed, which provided visual guidance. The caregiver was attuned to the person, fostering authentic collaboration, providing reassurance when needed, and allowing ample time for all aspects of the activity to take place. They stood near enough to help if required, while not standing over the person. There was unhurried silence when the person was conducting the task.

##### Example 2: family setting observation

###### Person with dementia’s actions—focused engagement

**Figure 5.**
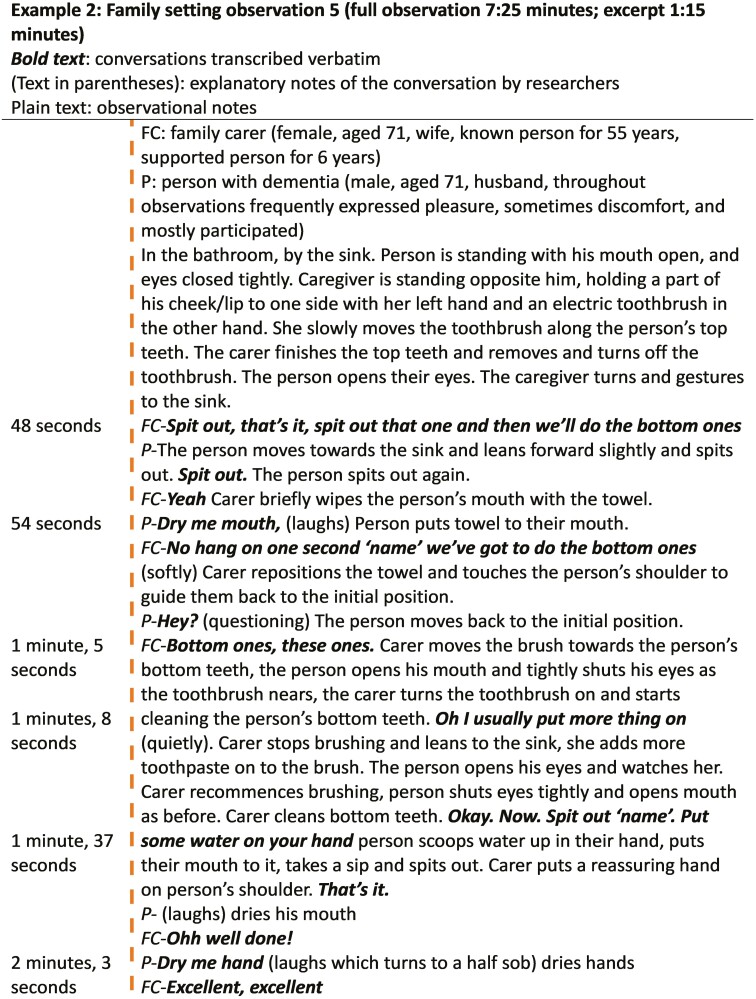
Observation Transcript 2, family setting.

Example 2 shows the person with dementia was engaged throughout the interaction (actively participating and watching the caregiver; see Figure 5). The person did not provide explicit consent but acted swiftly to comply with the caregiver’s directions (“*Spit out*”). The person stated what he was doing when he was doing it, for example, using “*Spit out*” and “*Dry me mouth*,” commentary for his actions. The person moved onwards with the task by drying his mouth. While drying his mouth, he laughed momentarily for no discernible reason, possibly due to being observed. When asked to wait due to bottom teeth needing cleaning, the person checked with the caregiver (“*hey?*”) to seek understanding of what needed to happen. The person complied with the caregiver guidance, as they moved back to the teeth cleaning position. They opened their mouth, then closed their eyes due to toothbrush going into their mouth. The person then rinsed their mouth as requested by the caregiver and dried their mouth and hand without prompt.

###### Caregiver’s actions—nurturing attentiveness

In Example 2, the caregiver guided the person through the activity in several ways; verbally introducing the next step of the activity in short, clear sentences (prompts) and when required, gently physically guiding the person into a suitable space for it to take place. The caregiver emanated a sense of togetherness, using the word “*we*” when introducing the next steps (“*then we’ll do the bottom ones*” “*we’ve got to do the bottom ones*”), demonstrating that they and the person with dementia were working as one toward a common goal. However, this communication feature could be regarded as an overinclusive “we,” which could pressure the person. In this interaction the person showed no negative response.

When the person started to deviate from the caregiver’s next expected step by drying his mouth, she softly redirected him to the second part of the activity verbally and nonverbally with a guiding hand. An instruct–endorse pattern (“*Spit out*”–“*that’s it*” and “*Put some water on your hand*”–“*That’s it*”) was used. The caregiver was attuned with the pace of the person, not rushing them, but also not allowing long pauses, which may have meant the person would have lost focus. Teeth cleaning happened slightly away from the sink to enable the caregiver enough room to get into a position opposite the person. After the activity, the caregiver put the person at their ease with praise (“*Well done*” and “*Excellent*”).

#### “Contentious” interactional components

“Contentious” components of care interactions were not common but were characterized by the person with dementia tolerating uncomfortable care or struggling to be heard. Caregivers had difficulties interpreting the situation, and sometimes pressed on with the activity despite having no clear signals to do so. We use an example from a family setting (Example 3) to demonstrate this category; however, we found the same features in care-home observations:

##### Example 3: family setting observation

###### Person with dementia’s actions: uneasiness

**Figure 6.**
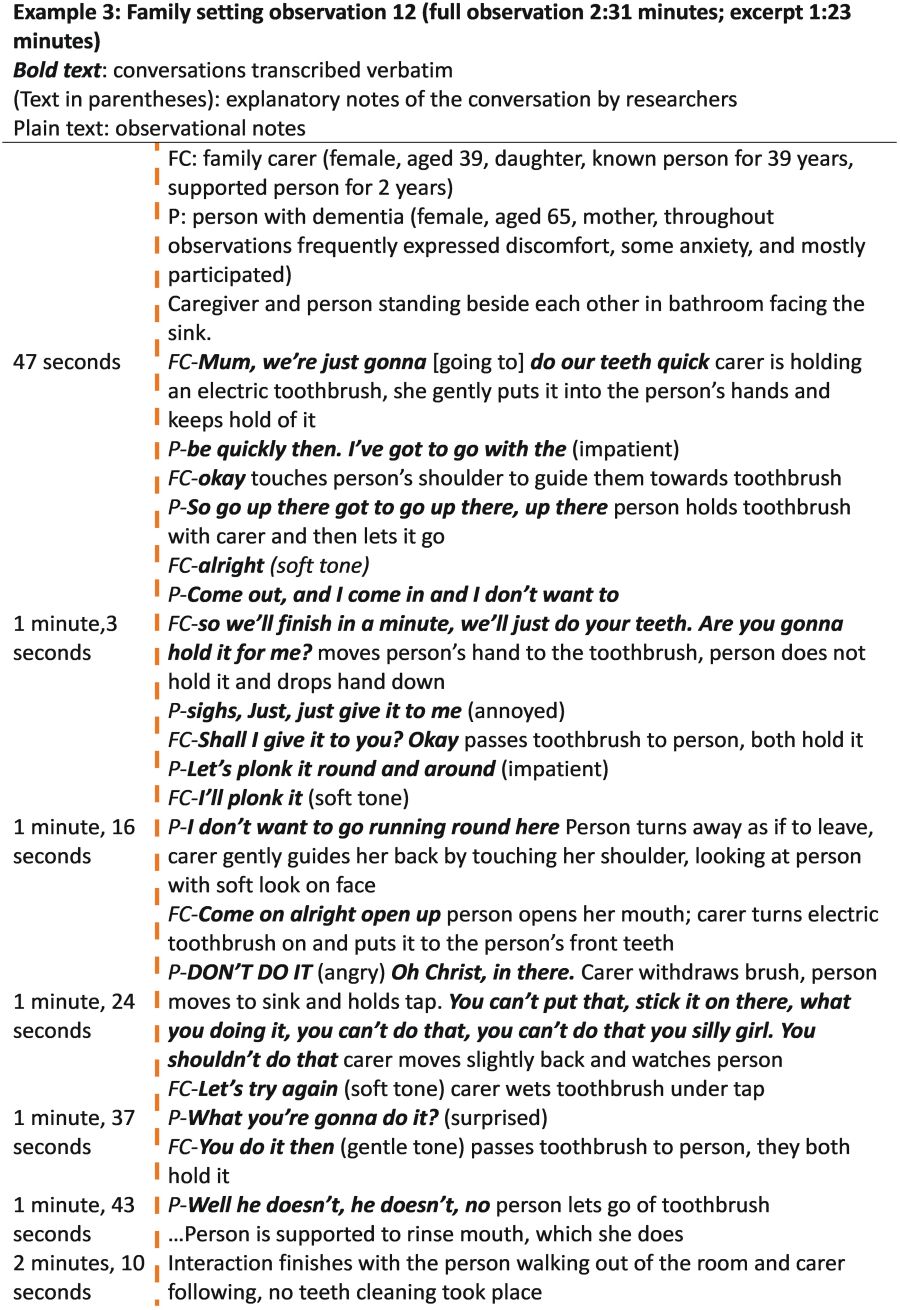
Observation Transcript 3, family setting.

Example 3 shows that throughout this interaction the person with dementia was uneasy (see Figure 6). The person indicated impatient assent saying, “*be quickly then*” and “*just give it* [toothbrush] *to me*.” However, they repeatedly also indicated they were unhappy to have the care completed for instance they let go of the toothbrush twice and said “*I don’t want to*” twice even though their verbal communication was impaired. This all occurred before the caregiver started to clean the person’s teeth, subsequently the person became very angry and shouted, “*don’t do it*.” The person continually tried to assert themselves and refuse teeth cleaning. After 2 min of uneasiness, the person with dementia walked out, without their teeth cleaned.

###### Caregiver’s actions: flustered uncertainty

Example 3 shows the caregiver employed many nurturing attentiveness approaches to try to invite the person with dementia to take part in the activity, including encouraging the person to conduct the activity themselves, using a guiding hand, trying to put the person at their ease by reassuring, using the person’s name (“*Mum*”) and the words “*quick*” and “*just*” to make the sound of the task appear simpler, quicker, and smaller. However, in this situation, these words also served to hurry the person to proceed with the care activity. The use of an overinclusive “we” (“*we’re*” “*we’ll*”) may have also triggered the person’s unease. The caregiver also pressed on with the care activity when the person repeatedly indicated they were not keen to go ahead and would like to refuse.

Pressing on may have been the reason the person started giving mixed messages where they overall appeared to be refusing to take part in the activity, but also showed a few words (“*let’s plonk it round and around*”) and actions (briefly holding the toothbrush and opening mouth) that appeared to make the caregiver think the person had assented to the activity taking place. The caregiver tried to interpret the person’s communication and ascertain whether they were assenting or not to decide how to proceed in the moment. The caregiver, flustered, continued to attempt to complete the activity, despite the person’s palpable uneasiness.

## Discussion

This study used video-recorded observations to examine features of personal care interactions between people with advanced dementia and caregivers (care-home staff and family carers). Findings showed that people with advanced dementia can participate (actively or by watching) to a greater extent in care interactions than is typically assumed. Refusals of care were present in a third of observation sections. Caregivers from both settings employed multiple skills for nurturing the person through care interactions, empathetically demonstrating collaboration between the individuals. Typically, people with advanced dementia were adeptly involved in care interactions by caregivers seamlessly filling the spaces where the person’s physical or cognitive abilities restricted them. This enabled the person to engage as much as they could, which appeared to work to amplify their personhood.

Our findings provide a real-world empirically informed reinvigoration of the notion of person-centered care in advanced dementia. The findings do not reflect a clear tailoring of care approach to the individual’s preferences, rather they show an enhancement of the person with advanced dementia’s personhood by placing their relational, embodied, and emotional experience at the center of the care interaction. This was enacted through nurturing attentiveness, which involved caregivers providing a relational nurturing structure around the person through attuning to pace and space, and skillfully inviting, reassuring, and guiding the person through the interaction. The extant literature also echoes those aspects: by taking account of the person’s physical being (Dewing, 2008) and providing an authentic collaboration, the person can feel respected, valued, and enact meaningful engagement (Fazio et al., 2018), which could enhance personhood (Tieu et al., 2022).

Nurturing attentiveness being linked to successful personal care interactions provides direction for future research aiming to improve care interactions. It is likely that many of the components of nurturing attentiveness (see Figure 3) could be learnt, for example, the use of the instruct–endorse pattern, reassuring, permission-seeking, and positioning of the caregiver. Greater nurturing attentiveness during care interactions could lead to the person with advanced dementia being more relaxed and consequently, less refusals of care. However, whether these practices can be fostered in all settings and with all caregivers is unknown. Barriers to implementation could include time restrictions in care homes and stress levels in family carers.

Our analyses allowed us to examine features of personal care interactions between care-home staff and family carers and people with advanced dementia in two ways. Observational video coding enabled us to determine the frequency of people with dementia’s key actions regarding engagement and refusals of care and qualitative content analysis allowed us to examine interactional dynamics in depth. From our analyses it appeared that discomfort, anxiety/sadness, and refusals of care were linked. These actions frequently accompanied the uneasiness of people with dementia when tolerating uncomfortable care or struggling to be heard and the flustered uncertainty of caregivers. Additionally, people with dementia displaying some pleasure in observations were often showing focused engagement, actively participating in, or watching the care activity. Pleasure often occurred when caregivers were enacting nurturing attentiveness particularly when emphasizing relational aspects through inviting the person to be part of the interaction or putting them at their ease. Our two analyses add greater certainty to our findings, each validating the other regarding successful and contentious care components.

We found, contrary to media reports stigmatizing dementia care work (e.g., Gregory, 2022), most caregiver actions were unhurried and attuned to the person, providing a nurturing attentiveness around the person to support their needs. However, care approaches were blended, with nurturing skills such as reassuring and permission-seeking present in the interactions, which were predominantly contentious for caregivers and/or people with advanced dementia. Contrarywise, in some predominantly successful interactions, caregivers could be overly nurturing and use overinclusive language (Shaw & Gordon, 2021). Adopting reassuring words and inclusive language may be appropriate for some people in advanced stages of dementia. For example, in our data the use of “for me” and “we” in some observations appeared to reflect caregivers presenting togetherness and was taken positively by the person. However, the use of these terms can be overinclusive, creating a dilemma; alternatives are “I” or “you” and may reflect an approach of *doing to* rather than *doing with*. Here, caregivers must balance the aim of portraying shared goals and togetherness against the risk of patronizing and alienating the person with elderspeak.

Past research has reinforced Kitwood’s (1997) notions of positive person work and malignant social psychology by showing caregiver actions and words can reduce, or lead to, agitation in people with dementia during personal care interactions (Williams & Herman, 2011). Our data supported this, with nurturing attentiveness allowing people with dementia to flourish and flustered uncertainty making them uneasy. Conversely, people with dementia’s refusals or mixed messages could lead to caregivers becoming flustered; therefore, in these situations each person’s state influenced the other’s. Positive communication features such as recognition, validation, and facilitation (Mundadan et al., 2023) were common in our data. However, there was a noticeable absence of negotiation (consulting on preferences and needs), which may have been due to caregivers’ existing knowledge of care recipients’ preferences or of their impaired communication ability due to advanced dementia (Mitchell, 2015) making negotiation difficult.

Our study included both family and care-home caregivers and has shown how similar the two groups are. Similarities in formal and informal caregiver understandings have been reported before (Herron & Wrathall, 2018). We did not set out to compare the two groups; instead, we aimed to examine features of varied personal care interactions. In doing so we were able to discern successful and contentious components of care interactions not bound by caregiver type. There were no clear differences depending on the care activity; however, activities requiring more cognitive processing and intrusion such as teeth cleaning or assisted eating may have elicited more refusals, and activities requiring less cognitive processing, for example, nail cutting or foot spa, may have elicited less participation from the person with dementia. Intimate care activities, such as going to the toilet, were not observed in our study, although ethnographic researchers have negotiated standing outside the door to listen to these interactions (Thompson et al., 2021). The main difference in our observations was that more pleasure was shown by people with dementia in care-home settings than in family settings (Figure 2). This appeared due to care-home participants (resident and caregiver) making efforts to socialize with people they did not see all the time, compared to family carers who were often caring for the person every day.

### Implications for Practice

Our analyses detailed real-world examples of what person-centered care may look like in practice. Caregivers of people with advanced dementia should consider their key role in either nurturing or making the person feel uncomfortable when assisting with personal care. To optimize personhood in personal care interactions with people with advanced dementia, caregivers should aim to nurture the relational and emotional experience of the person throughout the care interaction by attuning to the person’s pace and space, and inviting, reassuring, and guiding them through. Future research should consider these factors in the development of interventions to educate caregivers of people with advanced dementia.

### Strengths and Limitations

This study has drawn on data from real-world settings. Strengths of this study included the use of video recordings, which provided the opportunity to reexamine and verify the interactions in detail. This study examined both formal and informal caregiver groups; therefore, illuminating successful and contentious care components that go beyond one care setting. Observations were of different care activities, at different settings and of people at different stages of advanced dementia (severe, very severe, and profound).

We only have insight into those participants who agreed to take part who are likely to have positive views and/or experiences of their care actions. Purposive sampling enabled further variations to be present; however, scope was limited to those who had opted into the possibility of an observation. All caregivers were female and White British; therefore, the sample is not likely have been typical. As such, the dynamics recognized may or may not be part of a larger representative sample. We also only observed activities where the person was covered, excluding more intimate activities such as bathing or going to the toilet. It could be that the experience of personal care in such situations would further heighten the emotional impact of the interaction. Nevertheless, clear interpersonal components were still evident across these varied data, indicating that findings are likely to be transferable. The sample size for this exploratory study, although small, enabled the detailed examination of over 3-hr of video-recorded footage of otherwise private interactions. Caregivers may have altered their behavior due to the knowledge they were being recorded. However, they could not have created novel behavior outside their possible repertoire in the observed period. Additional insights may have been gained if observing at more than one time point. Close inspection of individual situations, such as in this study, cannot take account of complex wider circumstances that constrain care provided for people with dementia such as family carer fatigue or care staff time constraints.

## Conclusion

This is one of few empirical studies unpacking real-world observations to provide a rich understanding of interactions between a person living with dementia and those who provide care in formal and informal care settings. Our study contributes much needed insight into ways to improve personal care interactions for people with advanced dementia and caregivers. Findings suggest personal care interactions can be key moments for engagement and person-centered care. Caregivers’ relational communication, when enacting nurturing attentiveness, has potential to enhance a person with advanced dementia’s personhood. Conversely, caregiver actions representing flustered uncertainty in personal care interactions could be difficult for the person with dementia to cope with. To optimize person-centeredness in personal care interactions with people with advanced dementia, caregivers should aim to nurture the relational and emotional experience of the person throughout the care interaction by attuning to the person’s pace and space, and inviting, reassuring, and guiding them through.

## Supplementary Material

gnae004_suppl_Supplementary_Material

## Data Availability

Raw data cannot be shared due to ethical restrictions; however, anonymized transcripts are available on reasonable request from the corresponding author. This study was not preregistered.
