# Supplementary material for: Nurturing Attentiveness: A Naturalistic Observation Study of Personal Care Interactions Between People With Advanced Dementia and Their Caregivers
Source: Gerontologist. 2024 Jan 24;64(6):gnae004. doi: 10.1093/geront/gnae004 (PMC11102004; doi:10.1093/geront/gnae004)

**Online Supplementary Material**

| **Table A: Observations, interaction type and scale scores** | | | | | | |  |  |  |
| --- | --- | --- | --- | --- | --- | --- | --- | --- | --- |
| **Dyad number** | **Observation**  **Number / setting** | **Observation activity/ies** | **Observation duration**  **min.sec** | **Analysis 5-minute sections** | **Discomfort present** | **Pleasure** | **Anxiety/sadness** | **MPES predominant engagement** | **RTC-DAT score** |
| Family settings | | | | | | | | | |
| 1 | 1 Family | Beard combing | 00.44 | 00.00-00.44 | No | Some | None | Participated | 0 |
|  | 2 Family | Outer dressing | 01.25 | 00.00-01.25 | No | None | None | Participated | 0 |
| 2 | 3 Family | Morning routine: Face, teeth, hair | 06.19 | 00.00-05.00 | No | None | None | Participated | 0 |
| 3 | 4 Family | Nail cutting | 10.24 | 00.00-05.00  05.00-10.00 | No  Yes | Some  None | None  None | Watched  Watched | 0  0 |
| 4 | 5 Family | Shaving and teeth | 07.25 | 00.00-05.00 | Yes | Some | None | Participated | 1 |
|  | 6 Family | Nail cutting | 01.36 | 00.00-01.36 | Yes | Some | None | Other things | 2 |
|  | 7 Family | Outer dressing | 00.29 | 00.00-00.29 | No | Some | None | Participated | 0 |
|  | 8 Family | Hearing aids | 04.20 | 00.00-04.20 | No | Some | None | Watched | 0 |
|  | 9 Family | Medication | 00.29 | 00.00-00.29 | No | Some | None | Participated | 0 |
| 5 | 10 Family | Assisted eating | 36.33 | 00.00-05.00  05.00-10.00  10.00-15.00  15.00-20.00  20.00-25.00  25.00-30.00  30.00-35.00 | Yes  No  No  No  No  No  No | None  None  None  None  None  None  None | Some  None  None  None  None  None  None | Participated  Participated  Participated  Participated  Participated  Participated  Participated | 4  1  1  0  0  1  0 |
|  | 11 Family | Nail cutting | 09.21 | 00.00-05.00  05.00-09.21 | Yes  Yes | None  None | None  None | Other things  Stared into space | 0  3 |
|  | 12 Family | Teeth cleaning | 02.31 | 00.00-02.31 | Yes | None | Lots | Other things | 12 |
| Care-home settings | | | | | | | | | |
| 6 | 1 Care home | Teeth cleaning | 01.49 | 00.00-01.49 | Yes | None | Some | Participated | 10 |
|  | 2 Care home | Shaving | 02.17 | 00.00-02.17 | Yes | None | Some | Other things | 10 |
| 7 | 3 Care home | Nail cutting | 25.03 | 00.00-05.00  05.00-10.00  10.00-15.00  15.00-20.00  20.00-25.00 | Yes  Yes  Yes  Yes  Yes | None  None  Some  None  Some | None  None  None  None  None | Watched  Watched  Watched  Watched  Watched | 1  2  1  0  1 |
| 8 | 4 Care home | Nail cutting | 20.21 | 00.00-05.00  05.00-10.00  10.00-15.00  15.00-20.00 | No  No  No  No | None  Some  Some  Some | None  None  None  None | Participated  Participated  Participated  Participated | 0  0  0  0 |
| 9 | 5 Care home | Foot spa | 09.39 | 00.00-05.00  05.00-09.39 | No  No | Some  None | None  None | Participated  Watched | 0  0 |
| 10 | 6 Care home | Shaving | 04.30 | 00.00-04.30 | No | None | None | Participated | 0 |
|  | 7 Care home | Hair styling | 00.26 | 00.00-00.26 | No | None | None | Participated | 0 |
|  | 8 Care home | Teeth cleaning | 02.01 | 00.00-02.01 | No | Some | None | Participated | 0 |
| 11 | 9 Care home | Shaving | 02.32 | 00.00-02.32 | No | Some | None | Participated | 0 |
| 12 | 10 Care home | Hearing aids | 00.59 | 00.00-00.59 | No | None | None | Other things | 0 |
|  | 11 Care home | Morning routine: Feet, teeth, hair, outer dressing | 11.54 | 00.00-05.00  05.00-10.00 | No  No | Some  None | None  None | Participated  Participated | 0  0 |
| 13 | 12 Care home | Morning routine: Face, teeth, outer dressing | 07.15 | 00.00-05.00 | No | Some | None | Participated | 0 |
| 14 | 13 Care home | Foot spa | 08.45 | 00.00-05.00  05.00-08.45 | No  Yes | Some  Some | None  None | Participated  Participated | 0  0 |
|  | 14 Care home | Hair wash and dry | 05.43 | 00.00-05.00 | Yes | Some | None | Participated | 0 |
| **Overall total: 14 dyads** |  | **Overall total: 26 separate observation recordings** |  | **Overall total: 44 observation sections** |  |  |  |  |  |
| RCT-DAT Score: Frequency x Severity=Total; MPES: Menorah Park Engagement Scale | | | | | | | | | |

Figure A: Percentage of refusals of care present by setting.


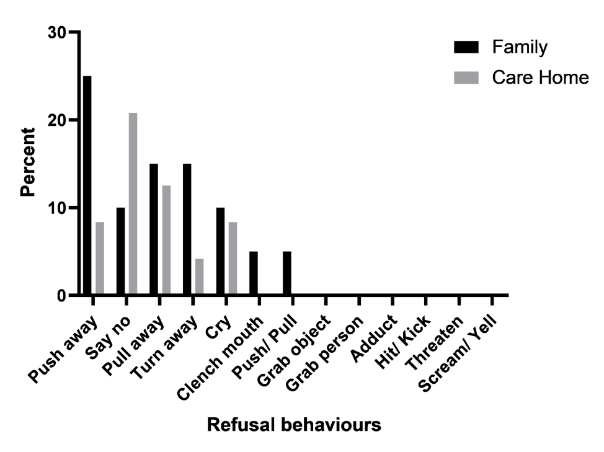

Supplement: gnae004_suppl_Supplementary_Material [file gnae004_suppl_supplementary_material.docx]
